# Arginine glycosylation enhances methylglyoxal detoxification

**DOI:** 10.1038/s41598-021-83437-0

**Published:** 2021-02-15

**Authors:** Samir El Qaidi, Nichollas E. Scott, Philip R. Hardwidge

**Affiliations:** 1 College of Veterinary Medicine, Kansas State University, Manhattan, KS 66506 USA; 2 Department of Microbiology and Immunology, University of Melbourne Within the Peter Doherty Institute for Infection and Immunity, Melbourne, 3000 Australia

**Keywords:** Microbiology, Bacteriology, Glycobiology

## Abstract

Type III secretion system effector proteins have primarily been characterized for their interactions with host cell proteins and their ability to disrupt host signaling pathways. We are testing the hypothesis that some effectors are active within the bacterium, where they modulate bacterial signal transduction and physiology. We previously determined that the *Citrobacter rodentium* effector NleB possesses an intra-bacterial glycosyltransferase activity that increases glutathione synthetase activity to protect the bacterium from oxidative stress. Here we investigated the potential intra-bacterial activities of NleB orthologs in *Salmonella enterica* and found that SseK1 and SseK3 mediate resistance to methylglyoxal. SseK1 glycosylates specific arginine residues on four proteins involved in methylglyoxal detoxification, namely GloA (R9), GloB (R190), GloC (R160), and YajL (R149). SseK1-mediated Arg-glycosylation of these four proteins significantly enhances their catalytic activity, thus providing another important example of the intra-bacterial activities of type three secretion system effector proteins. These data are also the first demonstration that a *Salmonella* T3SS effector is active within the bacterium.

## Introduction

Methylglyoxal (MGO) is a highly-reactive dicarbonyl compound that functions as a glycating agent that damages DNA and proteins^1,2^. Bacterial MGO detoxification systems generally consist of two independent but complementary pathways^3^. The glutathione-dependent pathway includes GloA, GloB, and GloC. GloA is a type I glyoxalase that catalyzes the conversion of hemithioacetal into *S*-lactoylglutathione. *S*-Lactoylglutathione is converted into d-lactate by the glyoxalase II isomers GloB and GloC^4–7^. The second pathway, which is glutathione-independent, is catalyzed by members of the DJ-1 superfamily such as YajL, Hsp31, YhbO, and ElbB^8–10^. Additional enzymes are also important to MGO detoxification, including Lgl, a metalloprotein with l-glutathione lyase activity^11^, DkgA, a protein with MGO reductase and beta-keto ester reductase activities^12,13^, the NADPH-dependent aldehyde reductase YqhD^14^, and the NADPH-dependent aldo–keto reductase YdiH^15^.

We and others have described the mechanism and importance of a conserved family of type III secretion system (T3SS) effectors named NleB in both *Escherichia coli* and *Citrobacter rodentium* and SseK in *Salmonella enterica*^16–19^. Most *E. coli* strains encode two NleB proteins (NleB1 and NleB2), while *Salmonella* encodes three SseK proteins (SseK1, SseK2, SseK3)^20^. These effectors are glycosyltransferases that modify host protein substrates with *N*-acetyl glucosamine (GlcNAc) on specific arginine residues^18^. Crystal structures of several NleB/SseK orthologs show a high degree of structural similarity and consist of three major domains; a catalytic domain including the essential DXD and HEN (His–Glu–Asn) motifs, a helix–loop–helix (HLH) domain, and a C-terminal lid domain which is also required for the catalytic activity of the enzyme^18,21,22^.

Arginine glycosylation is unusual because it occurs on the guanidinium groups of arginines, which are poor nucleophiles. NleB glycosyltransferase activity is essential to bacterial virulence^16^. Multiple host protein substrates for the NleB/SseK orthologs have been described and include the Fas-associated protein with Death Domain (FADD), tumor necrosis factor receptor type 1-associated death domain protein (TRADD), and the receptor interaction serine/threonine-protein kinase 1 (RIPK1)^17^. NleB1 disrupts tumor necrosis factor receptor (TNFR)-associated factor (TRAF) signaling, leading to inhibition of the pro-inflammatory NF-κB pathway^16,17,19^. *E. coli* NleB1 glycosylates arginine residues in the death domains of FADD, TRADD, RIPK1, and TNFR1^17,19^. *Salmonella* SseK1 glycosylates TRADD^17^, SseK2 glycosylates FADD, and SseK3 glycosylates TNFR1 and TRAILR^23^, as well as the small GTPase Rab1^24^. *C. rodentium* NleB, *E. coli* NleB1, and *Salmonella* SseK1 also glycosylate glyceraldehyde 3-phosphate dehydrogenase (GAPDH)^16,25^.

Most previous research with NleB and SseK has focused on their inhibition of proteins involved in the host innate immune response^16–19^. We previously observed that NleB also glycosylates the *C. rodentium* glutathione synthetase (GshB)^26^. This intra-bacterial Arg-glycosylation contributes to bacterial survival in hydrogen peroxide stress conditions by enhancing GshB activity and increasing intracellular levels of glutathione (GSH). Thus, NleB is active and performs important biological functions within the bacterium, prior to its secretion. Here we investigated the potential intra-bacterial activities of the *Salmonella* SseK enzymes. We found that SseK1 is active within *Salmonella enterica* and that it glycosylates GloA, GloB, GloC, and YajL on arginine residues to significantly enhance their enzymatic activities and provide increased resistance to MGO.

## Results

### SseK1 and SseK3 affect methylglyoxal (MGO) resistance

**Figure 1:**
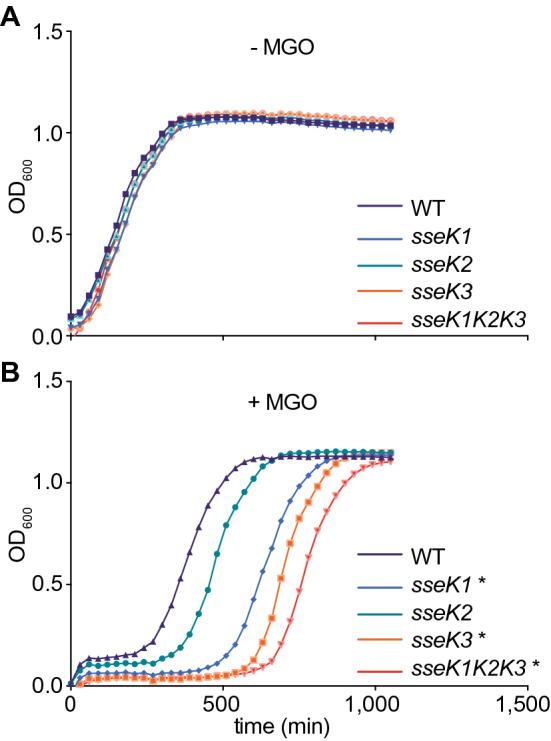
Bacterial growth assays. (**A**) *S. enterica* growth (OD_600_) as a function of time (min) in the absence of 1.0 mM MGO. (**B**) *S. enterica* growth in the presence of 1.0 mM MGO. Asterisks indicate significantly different growth rates as compared to the wild-type strain.

We previously showed that *C. rodentium* NleB promotes bacterial survival in the presence of hydrogen peroxide by glycosylating the glutathione synthetase enzyme GshB^26^. By contrast, *Salmonella* is resistant to hydrogen peroxide due to the activity of redundant hydrogen peroxide scavengers^27^. Here we asked whether the NleB orthologs in *Salmonella*, SseK1, SseK2, and SseK3, might protect *Salmonella* from other forms of chemical stress. We compared the growth rates of *Salmonella* strains possessing or lacking SseK orthologs in the presence or absence of 1 mM MGO. Both the *sseK1* and *sseK3* mutants had a significant growth defect in the presence of MGO, despite having similar growth rates in the absence of MGO, whereas the growth rate of the *sseK2* mutant was not significantly different as compared to the wild-type strain (Fig. 1). These data suggest a role for SseK1 and SseK3, but not SseK2 in MGO detoxification.

### SseK1 glycosylates MGO detoxification enzymes

**Figure 2:**
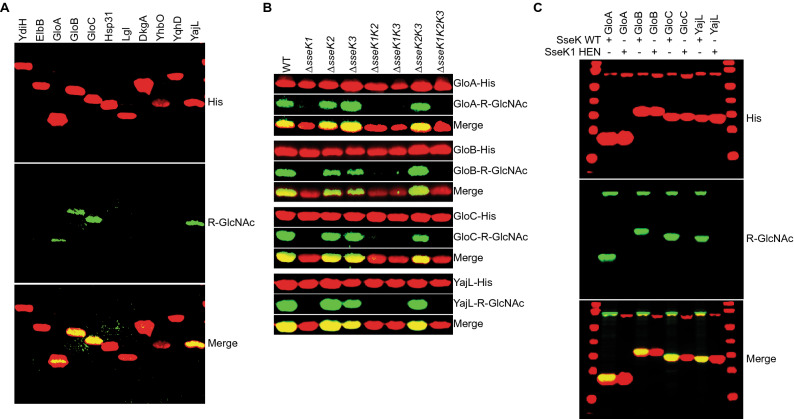
SseK glycosylates multiple MGO detoxification enzymes. (**A**) Western blot analysis of indicated proteins implicated in MGO detoxification for in vivo Arg-glycosylation in *S. enterica*. (**B**) In vivo Arg-glycosylation of GloA, GloB, GloC, and YajL as a function of their co-expression with SseK1, SseK2, and/or SseK3. Original blots are shown in Supplemental Figure S1. (**C**) In vitro Arg-glycosylation of GloA, GloB, GloC, and YajL after incubation with SseK1 WT or HEN mutant proteins.

SseK1 and SseK3 are arginine-specific glycosyltransferases^18^ and *Salmonella* strains lacking *sseK1* or *sseK3* are hypersensitive to MGO (Fig. 1). We tested the hypothesis that SseK1 and/or SseK3 glycosylate one or more *Salmonella* enzymes involved in MGO detoxification to regulate their activity. To do this, we cloned and expressed in *Salmonella* recombinant forms of the DJ-1 superfamily members YajL, Hsp31, YhbO, and ElbB, the glyoxalases GloA, GloB, and GloC, the l-glutathione lyase Lgl, the methylglyoxal reductase DkgA, the aldo–keto-reductase YdiH, and the putative aldehyde reductase YqhD. We then immunoblotted protein lysates for R-GlcNAc on each protein and observed that the type I glyoxalase GloA, the type II glyoxalase isomers GloB and GloC, and the deglycase YajL were glycosylated on an arginine residue in vivo (Fig. 2A).

We then repeated the glycosylation assays in *Salmonella* strains possessing or lacking each possible combination of SseK1, SseK2, and SseK3 and concluded that SseK1 glycosylates GloA, GloB, GloC, and YajL, as Arg-glycosylation was absent in any strain lacking SseK1 but was present independently of SseK2 and SseK3 (Fig. 2B). To further corroborate these data, we performed in vitro glycosylation assays using recombinant enzymes and substrates. All substrates were glycosylated by WT SseK1, but not by an SseK1 (HEN) mutant^18^ which lacks glycosyltransferase activity (Fig. 2C).

### SseK1-mediated glycosylation increases the activity of GloA, GloB, GloC, and YajL

**Figure 3:**
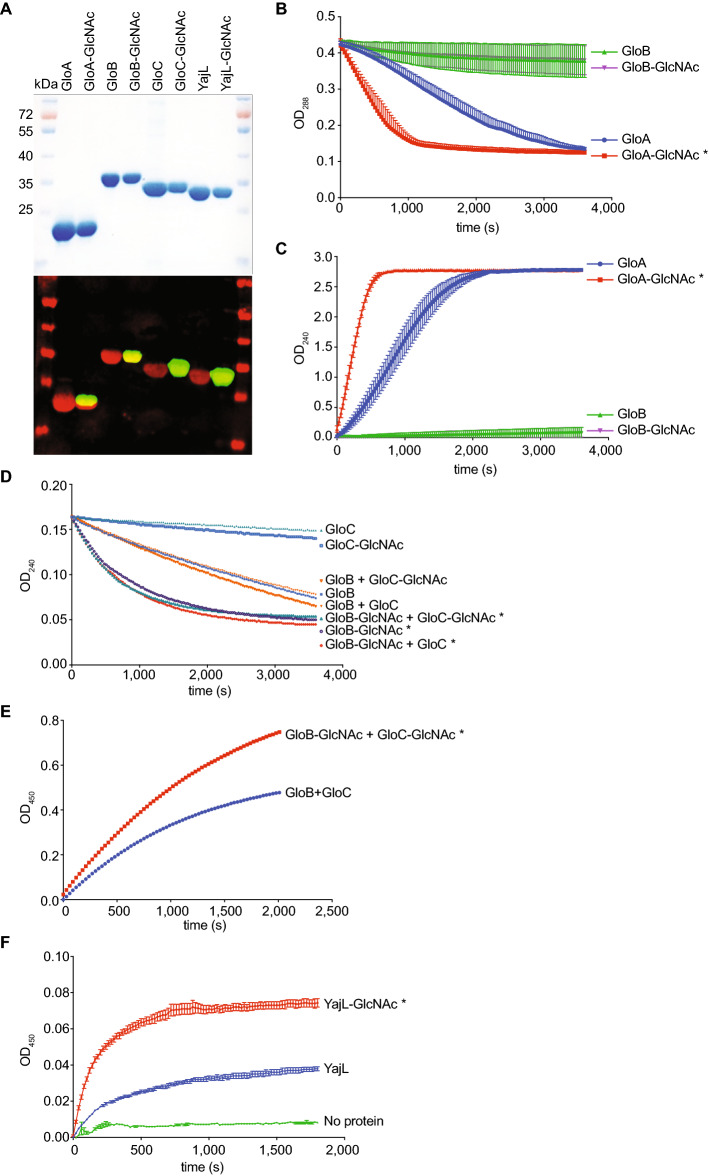
In vitro enzyme assays. (**A**) Purification of as glycosylated or unglycosylated forms of GloA, GloB, GloC, and YajL after their co-expression with SseK1. Western blotting was used to confirm the protein glycosylation state (red, anti-His; green, ant-R-GlcNAc). (**B-C**) GloA activity assays. (**B**) Catalysis of *S*-lactoylglutathione consumption as a function of GloA glycosylation was monitored as a function of time. (**C**) *S*-Lactoylglutathione production as a function of GloA glycosylation was monitored as a function of time. (**D,E**) Glyoxalase II assays. (**D**) Catalysis of *S*-lactoylglutathione consumption as a function of GloB-GloC glycosylation. (**E**) d-lactate production as a function of GloB-GloC glycosylation. (**F**) YajL repair assay; GAPDH activity was monitored as a function of YajL glycosylation state. Asterisks in panels indicate significantly reaction rates between glycosylated and unglycosylated enzymes.

We hypothesized that SseK1 glycosylation of GloA, GloB, GloC, and YajL might affect the respective activity of these enzymes, consistent with the *sseK1* growth phenotype in MGO. We purified GloA, GloB, GloC, and YajL in their glycosylated or unglycosylated forms by co-expressing them (or not) with SseK1 (Fig. 3A, top), verified their glycosylation state using immunoblotting (Fig. 3A, bottom), and then used these recombinant proteins in enzyme assays. GloA is a type I glyoxalase that uses glutathione as a cofactor to convert hemithioacetal, which absorbs at OD_288_, into *S*-lactoylglutathione, which absorbs at OD_240_^4^. We assayed the activity of GloA as a function of its glycosylation state and observed a significantly enhanced activity in comparison to the native enzyme (Fig. 3B), as measured by a significant increase in the rate of hemithioacetal consumption. To corroborate these data, we also quantified the production of *S*-lactoylglutathione and observed a corresponding increase in *S*-lactoylglutathione production due to GloA glycosylation (Fig. 3C). We used GloB as a control in these assays and, as expected, observed no significant activity.

GloB and GloC are glyoxalase II isomers that catalyze the conversion of *S*-lactoylglutathione into d-lactate^28^. GloB is described as the major glyoxalase II, while GloC has a minor glyoxalase II activity, with maximal activity is reached by a combination of GloB and GloC^7^. We performed a glyoxalase II assay to compare the activity of individual or a combination of glycosylated and unmodified forms of GloB and GloC (Fig. 3D). As reported previously^7^, GloB was more active than GloC (Fig. 3D). SseK1-mediated glycosylation of GloB and GloC significantly enhanced their activity (Fig. 3D), as measured by a significant increase in the rate of *S*-lactoylglutathione consumption, as well a significant increase in the rate of d-lactate production (Fig. 3E).

YajL is a deglycase that repairs MGO-damaged proteins^8^. We measured the capacity of YajL to repair MGO-mediated glycated GAPDH, by performing GAPDH activity assays as a function of YajL glycosylation state. The glycosylated form of YajL was significantly more active than the native form of YajL, as measured by an increase in the activity of repaired GAPDH as a function of YajL glycosylation (Fig. 3F). Taken together, these data show that SseK1-mediated glycosylation of the glyoxalases GloA, GloB, and GloC, as well as the deglycase YajL increases their enzymatic activities, which explains, at least in part, the reduced growth of the *sseK1* mutant in comparison to the parental strain in the presence of MGO.

### Glycosylation site mapping

**Figure 4:**
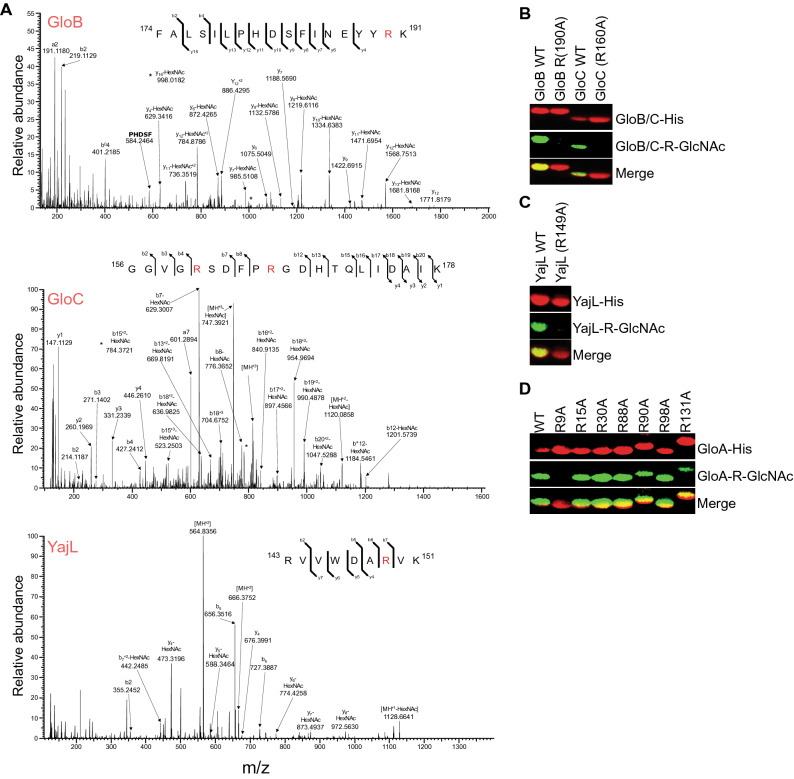
Glycosylation sites. (**A**) HCD spectra of the in vivo glycosylated GloB, GloC and YajL peptides ^174^FALSILPHDSFINEYYRK^191^, ^156^GGVGESDFPRGDHTQLIDAIK^178^, and ^143^RVVWDARVK^151^ confirms glycosylation is localized to R190, R160, and R149, respectively. (**B**) Western blot analysis of in vitro GloB and GloC glycosylation assays. (**C**) Western blot analysis of in vitro YajL glycosylation assays. (**D**) Site-directed mutagenesis and Western blot analysis to map the GloA glycosylation site to R9.

We used mass spectrometry and site-directed mutagenesis to identify the glycosylation sites on each protein substrate. We determined by using mass spectrometry that SseK1 glycosylates GloB on R190, GloC on R160 or R165, and YajL on R149 (Fig. 4A). We corroborated the mass spectrometry data by performing R-GlcNAc specific immunoblots with wild-type and mutant proteins, confirming these sites and the modification of R160 on GloC (Fig. 4B,C). Although GloA was examined using both Lys-C and trypsin digestion, we were unable to achieve complete coverage of all arginine residues within this protein. Within GloA, multiple arginines are flanked by lysine residues, which, when cleaved by Lys-C or trypsin, result in short (< 6 AAs) peptides which are poorly amenable to LC–MS analysis. Because a GloA peptide could not be generated for MS/MS analysis, we instead used site-directed mutagenesis to localize the glycosylation site on this protein and found that SseK1 glycosylates GloA on R9 (Fig. 4D).

## Discussion

**Figure 5:**
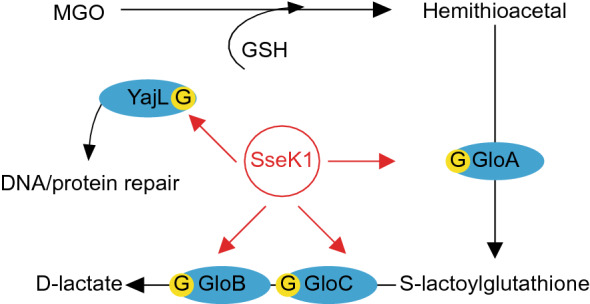
Working Model. SseK1 glycosylates GloA, GloB, GloC and YajL. GloA glycosylation enhances the conversion of hemithioacetal to *S*-lactoylgluatathione. GloB and GloC glycosylation enhances the conversion of *S*-lactoylgluatathione to d-lactate. YajL glycosylation enhances deglycase protein repair activities.

This study was undertaken to investigate the potential role of *Salmonella* T3SS effectors in providing resistance to methylglyoxal and to determine whether the NleB orthologs SseK1, SseK2, and SseK3 are active within *Salmonella*. Our data show that GloA, GloB, GloC, and YajL are glycosylated on specific arginine residues by SseK. Such glycosylation enhances resistance to MGO and also enhances the repair of MGO-damaged proteins (Fig. 5). Although both the s*seK1* and *sseK3* mutants had a growth phenotype in the presence of MGO (Fig. 1), we did not detect any *Salmonella* glycosylation targets for SseK3. Possible explanations include either the relatively small number of potential substrates we characterized in this study or the possibility that SseK3 might bind a bacterial substrate without glycosylating it, as seen for the host protein TRIM32^29^. We did not observe a role for the SseK enzymes in mediating *Salmonella* resistance to either hydrogen peroxide or glyoxal (data not shown).

The importance of MGO detoxification to bacterial virulence has been reported in several other systems, including *Burkholderia pseudomallei* and Group A Streptococcus (GAS)^30,31^. Over-expression of *B. pseudomallei* BPSS2242 in *E. coli* increases bacterial survival in the presence of MGO via an NADPH-dependent reductase activity^30^. Deleting GloA from GAS sensitizes GAS to MGO and increases its susceptibility to killing by human neutrophils^31^. In *Salmonella*, the lactoylglutathione lyase Lgl is known to also be important for MGO detoxification^11^, but we did not detect Lgl as an SseK1 substrate.

Similar to our previous study with NleB-mediated enhancement of glutathione synthetase activity in *C. rodentium*^26^, it remains to be determined why Arg-glycosylation affects the activity of the GloABC and YajL enzymes in *Salmonella*. The glycosylated arginines are distant from the enzyme active sites^10,32,33^; thus, activation might be the result of the post-translational modification mimicking an allosteric modification of the enzymes. Overall, these data reinforce the notion that T3SS effectors are active within bacterial cells and their activity appears to be tightly integrated with bacterial physiology, in this case, the ability to resist exogenous stressors. These data are also the first demonstration that a *Salmonella* T3SS effector is active within the bacterium.

Our work prompts several important questions that remain to be answered regarding the apparent dual activity of the NleB/SseK T3SS effectors within both the bacterium and the host cell. T3SS effectors are typically bound by a cognate chaperone to be guided to the injectisome complex^34^. This allows effectors to remain partially unfolded and competent for secretion through the secretion channel^35^. Some chaperones also prevent mis-targeting of effectors to bacterial membranes^36^ and inhibit their degradation by proteases^37^. No chaperone for SseK1 or NleB has been identified and it is currently unclear to what extent chaperone binding, or the lack thereof, may explain the intra-bacterial activity of the SseK1 we observe here and in earlier studies^26^.

We previously evaluated whether the multi-cargo chaperone CesT affected *C. rodentium* NleB activity but found that NleB activity was independent of CesT^26^. Thus, it is unclear what role, if any, chaperones may play in regulating the folding state of SseK1 to allow the effector to function within *Salmonella* and then be secreted through the T3SS. If there is an NleB/SseK cognate chaperone, the effector may shuttle between folded (active) and unfolded (inactive) states as a function of its binding to the chaperone. It is conceivable that such an equilibrium might be affected by exogenous stressors (e.g. high MGO concentrations) requiring intra-bacterial effector activity. Such a scenario would likely require post-translational, rather than co-translational secretion of SseK1.

The percentage of NleB/SseK that is secreted and the percentage of NleB/SseK that remains within the bacterium is also unclear. While such concepts could theoretically be addressed by performing pulse-chase and single-cell imaging experiments, this issue remains beyond the scope of the experiments described here. Such host/bacterial effector ratios would conceivably be influenced by the environmental conditions the bacterium is facing, i.e. whether the bacterium is in a secretion-competent state. NleB expression is induced and secreted in conditions that permit the expression of the EspADB translocon^38^. In *Salmonella*, SseK1 is expressed in both SPI1- and SPI2-inducing conditions^39^, but is higher in SPI2-inducing conditions^40^. SseK2 and SseK3 are expressed in SPI2-inducing conditions and are dependent upon the SsrA/SsrB two-component regulatory system^20^.

It is clear from our studies here, as well as our previous study of *C. rodentium* NleB^26^, that the NleB/SseK orthologs function within both the bacterium and in the host cell after secretion through the T3SS. However, one potentially significant issue that may limit the broad impact of our data is that is generally unclear whether the activities of NleB/SseK are representative of the many other T3SS effectors. It is conceivable that NleB and other non-LEE encoded effectors might be less tightly transcriptionally regulated as compared to other effectors and might thus have a more constitutive expression profile. This class of effector may function normally inside the bacterium and their functions within host cells may be of secondary importance. In contrast, T3SS effectors whose expression is tightly coordinated with T3SS activation signals and whose secretion is strongly dependent upon cognate chaperones may indeed not be found to have intra-bacterial activity. To address such questions, we await the development of assays suitable for monitoring the potential intra-bacterial activities of other T3SS effectors with defined enzymatic activities.

## Materials and methods

### Plasmids, strains, and cloning

The plasmids and strains used in this study are listed in Tables 1 and 2, respectively. Wild-type *sseK1* (*Salmonella enterica*) and its derivative H244A E255A N256A, were cloned into pET42a. Wild-type *gloA, gloB, gloC,* and *yajL,* as well as their arginine/alanine derivatives were cloned into a modified pET28a vector (in which the T7 promoter was replaced with the *tac* promoter) by using the ABC cloning method^41^. Gene deletions were constructed using lambda red recombination with the pKD3 and pKD119 plasmids^42^. Protein purification was performed as described previously^26^.

**Table 1:** Plasmids used in this study.

Plasmid	Source
FLAG-SseK1	^18^
GST-SseK1	^25^
GST-SseK1 (H244A,E255A,N256A)	^18^
His-YajL	This study
His-GloA	This study
His-GloA (R9A)	This study
His-GloA (R15A)	This study
His-GloA (R30A)	This study
His-GloA (R88A)	This study
His-GloA (R90A)	This study
His-GloA (R98A)	This study
His-GloA (R131A)	This study
His-GloB	This study
His-GloB (R190A)	This study
His-GloC	This study
His-GloC (R160A)	This study
His-Lgl	This study
His-DkgA	This study
His-Hsp31	This study
His-YhbO	This study
His-ElbB	This study
His-YdiH	This study
His-YqhD	This study
His-YajL (R149A)	This study

**Table 2:** Strains used in this study.

Strain	Source
*E. coli* BL21(DE3)+pHis-YajL	This study
*E. coli* BL21(DE3)+pHis-GloA	This study
*E. coli* BL21(DE3)+pHis-GloB	This study
*E. coli* BL21(DE3)+pHis-GloC	This study
*E. coli* BL21(DE3)+GST-SseK1	^25^
*E. coli* BL21(DE3)+GST-SseK1 (H244A E255A N256A)	^18^
*Salmonella typhimurium* ATCC 14028	^47^
*S. typhimurium ΔsseK1*	^47^
*S. typhimurium ΔsseK2*	^47^
*S. typhimurium ΔsseK3*	^47^
*S. typhimurium ΔsseK1ΔsseK2*	^47^
*S. typhimurium ΔsseK1ΔsseK3*	^47^
*S. typhimurium ΔsseK2ΔsseK3*	^47^
*S. typhimurium ΔsseK1ΔsseK2ΔsseK3*	^47^
*S. typhimurium*+pHis-YajL	This study
*S. typhimurium ΔsseK1*+pHis-YajL	This study
*S. typhimurium ΔsseK2*+pHis-YajL	This study
*S. typhimurium ΔsseK3*+pHis-YajL	This study
*S. typhimurium ΔsseK1ΔsseK2*+pHis-YajL	This study
*S. typhimurium ΔsseK1ΔsseK3*+pHis-YajL	This study
*S. typhimurium ΔsseK2ΔsseK3*+pHis-YajL	This study
*S. typhimurium ΔsseK1ΔsseK2ΔsseK3*+pHis-YajL	This study
*S. typhimurium*+pHis-YajL (R149A)	This study
*S. typhimurium*+pHis-GloA	This study
*S. typhimurium ΔsseK1*+pHis-GloA	This study
*S. typhimurium ΔsseK2*+pHis-GloA	This study
*S. typhimurium ΔsseK3*+pHis-GloA	This study
*S. typhimurium ΔsseK1ΔsseK2*+pHis-GloA	This study
*S. typhimurium ΔsseK1ΔsseK3*+pHis-GloA	This study
*S. typhimurium ΔsseK2ΔsseK3*+pHis-GloA	This study
*S. typhimurium ΔsseK1ΔsseK2ΔsseK3*+pHis-GloA	This study
*S. typhimurium*+pHis-GloA (R9A)	This study
*S. typhimurium*+pHis-GloA (R15A)	This study
*S. typhimurium*+pHis-GloA (R30A)	This study
*S. typhimurium*+pHis-GloA (R88A)	This study
*S. typhimurium*+pHis-GloA (R90A)	This study
*S. typhimurium*+pHis-GloA (R98A)	This study
*S. typhimurium*+pHis-GloA (R131A)	This study
*S. typhimurium*+pHis-GloB	This study
*S. typhimurium ΔsseK1*+pHis-GloB	This study
*S. typhimurium ΔsseK2*+pHis-GloB	This study
*S. typhimurium ΔsseK3*+pHis-GloB	This study
*S. typhimurium ΔsseK1ΔsseK2*+pHis-GloB	This study
*S. typhimurium ΔsseK1ΔsseK3*+pHis-GloB	This study
*S. typhimurium ΔsseK2ΔsseK3*+pHis-GloB	This study
*S. typhimurium ΔsseK1ΔsseK2ΔsseK3*+pHis-GloB	This study
*S. typhimurium*+pHis-GloB (R190A)	This study
*S. typhimurium*+pHis-GloC	This study
*S. typhimurium ΔsseK1*+pHis-GloC	This study
*S. typhimurium ΔsseK2*+pHis-GloC	This study
*S. typhimurium ΔsseK3*+pHis-GloC	This study
*S. typhimurium ΔsseK1ΔsseK2*+pHis-GloC	This study
*S. typhimurium ΔsseK1ΔsseK3*+pHis-GloC	This study
*S. typhimurium ΔsseK2ΔsseK3*+pHis-GloC	This study
*S. typhimurium ΔsseK1ΔsseK2ΔsseK3*+pHis-GloC	This study
*S. typhimurium*+pHis-GloC (R160A)	This study
*S. typhimurium*+His-Lgl	This study
*S. typhimurium*+His-DkgA	This study
*S. typhimurium*+His-Hsp31	This study
*S. typhimurium*+His-YhbO	This study
*S. typhimurium*+His-ElbB	This study
*S. typhimurium*+His-YdiH	This study
*S. typhimurium*+His-YqhD	This study

### In vitro glycosylation assays

Assays were performed as described previously^25^. SseK1 (200 nM) was incubated with 1 μM of either wild-type or mutated forms of GloA, GloB, GloC or YajL in buffer containing 50 mM Tris–HCl pH 7.4, 1 mM UDP-GlcNAc, 10 mM MnCl_2_, and 1 mM DTT. After 2 h incubation at RT, samples were subjected to western blotting using an anti-R-GlcNAc monoclonal antibody (Abcam).

### Bacterial growth assays

One half of one percent of an overnight culture of *Salmonella* strains was used to inoculate 50 ml of LB medium in the presence or absence of 1 mM methylglyoxal. Bacterial growth was monitored for 16 h at 37 °C using an automated plate reader.

### GAPDH activity assay

Bacterial GAPDH was incubated overnight at room temperature in a buffer containing 100 mM NaH_2_PO_4_ pH 7.0, in the presence of 5 mM of MGO. Glycated GAPDH (200 nM) was then incubated with either 100 nM YajL, YajL-GlcNAc, or no enzyme. GAPDH activity was monitored using the GAPDH Activity Assay Kit (# MAK277, Sigma).

### Glyoxalase I activity assay

Glutathione (5 mM) was incubated with 5 mM MGO for 1 h at room temperature in 100 mM NaH_2_PO_4_ pH 7.0 to generate hemithioacetal. Hemithioacetal was then incubated with either 50 nM GloA or GloA-GlcNAc and both hemithioacetal consumption and *S*-lactoylglutathione production was measured as a function of time by monitoring OD_288_ and OD_240_, respectively, in an automated plate reader.

### Glyoxalase II activity assay

*S*-lactoylglutathione (1 mM) was incubated with 50 nM GloB, GloB-GlcNAc, GloC GloC-GlcNAc or combinations of either form of GloB and GloC in 100 mM NaH_2_PO_4_ pH 7.0. Glyoxalase II activity was monitored by quantifying the amount of *S*-lactoylglutathione remaining as a function of time by monitoring OD_240_ in an automated plate reader.

### Digest of gel-separated proteins

Affinity-purified proteins were separated using SDS-PAGE, fixed, and visualized with Coomassie staining. Bands of interest were excised and destained in a 50:50 solution of 50 mM NH_4_HCO_3_, 100% ethanol for 20 min at room temperature with shaking at 750 rpm. Destained samples were then washed with 100% ethanol, vacuum-dried for 20 min, and rehydrated in 50 mM NH_4_HCO_3_ and 10 mM DTT. Reduction was carried out for 1 h at 56 °C with shaking. The reducing buffer was then removed, and the gel bands were washed twice in 100% ethanol for 10 min to remove residual DTT. Reduced ethanol washed samples were sequentially alkylated with 55 mM iodoacetamide in 50 mM NH_4_HCO_3_ in the dark for 45 min at room temperature. Alkylated samples were then washed with two rounds of 100% ethanol and vacuum dried. Alkylated samples were then rehydrated with either 20 ng/µl of trypsin (Promega) or 20 ng/µl of Lys-C (Wako Chemicals) in 40 mM NH_4_HCO_3_ at 4 °C for 1 h. Excess trypsin was removed, gel pieces were covered in 40 mM NH_4_HCO_3_ and incubated overnight at 37 °C. Peptides were concentrated and desalted using C18 stage tips^43^ before analysis by LC–MS.

### Reverse phase LC–MS/MS

Peptide samples were resuspended in Buffer A* (2% MeCN, 0.1% TFA) and separated using a two-column chromatography set up composed of a PepMap100 C18 20 mm × 75 μm trap and a PepMap C18 500 mm × 75 μm analytical column (Thermo Fisher Scientific), similar to as described previously^26^. Samples were concentrated onto the trap column at 5 μl/min for 5 min with Buffer A (0.1% formic acid, 2% DMSO) then infused into a Q-Exactive plus Mass Spectrometer (Thermo Fisher Scientific) at 300 nl/minute via the analytical column using a Dionex Ultimate 3000 UHPLC (Thermo Fisher Scientific). Ninety five-minute analytical runs were undertaken by altering the buffer composition from 2% Buffer B (0.1% formic acid, 77.9% acetonitrile, 2% DMSO) to 28% B over 1 h, then from 28% B to 4% B over 10 min, then from 40% B to 100% B over 2 min. The composition was held at 100% B for 3 min, and then dropped to 2% B over 5 min before being held at 2% B for another 15 min. The Q-Exactive plus Mass Spectrometer was operated in a data-dependent mode, acquiring one full precursor scan (resolution 70,000; 375–1800 m*/z*, AGC target of 1 × 10^6^) followed by 10 data-dependent HCD MS–MS events (using three collision energies of 28, 35, and 40; resolution 35 k AGC target of 2 × 10^5^ with a maximum injection time of 110 ms).

### Mass spectrometry data analysis

Identification of Arg-glycosylation events was accomplished using MaxQuant (v1.6.3.4)^44^. The predicted amino acid sequences for GloA, GloB, GloC, and YajL were combined into a database with the Salmonella typhimurium SL1344 proteome (Uniprot accession: UP000008962) and searched, allowing carbamidomethylation of cysteine set as a fixed modification and the variable modifications of oxidation of methionine and Arg*-*GlcNAcylation (H_13_C_8_NO_5_; 203.0793 Da to Arginine). Searches were performed with either Trypsin or Lys-C cleavage specificity towards each protein sample, allowing 2 miscleavage events with a maximum false discovery rate (FDR) of 1.0% set for protein and peptide identifications. The resulting modified peptide output was processed within the Perseus (v1.4.0.6)^45^ analysis environment to remove reverse matches and common protein contaminants. The mass spectrometry proteomics data have been deposited to the ProteomeXchange Consortium via the PRIDE^46^ partner repository with the dataset identifier PXD021878.

### Statistical analyses

Bacterial growth assays were analyzed using non-linear regression followed by Dunn’s multiple comparison testing. Enzyme assays were analyzed using linear regression. P values < 0.05 were considered significant.

## Supplementary Information

Supplementary Information 1.

Supplementary Information 2.

## References

[1] Kalapos MP (1999). Methylglyoxal in living organisms: Chemistry, biochemistry, toxicology and biological implications. Toxicol. Lett..

[2] Thornalley PJ (1990). The glyoxalase system: New developments towards functional characterization of a metabolic pathway fundamental to biological life. Biochem. J..

[3] Lee C, Park C (2017). Bacterial responses to glyoxal and methylglyoxal: Reactive electrophilic species. Int. J. Mol. Sci..

[4] Clugston SL (1998). Overproduction and characterization of a dimeric non-zinc glyoxalase I from *Escherichia coli*: Evidence for optimal activation by nickel ions. Biochemistry.

[5] Clugston SL, Yajima R, Honek JF (2004). Investigation of metal binding and activation of *Escherichia coli* glyoxalase I: Kinetic, thermodynamic and mutagenesis studies. Biochem. J..

[6] Ozyamak E (2010). The critical role of *S*-lactoylglutathione formation during methylglyoxal detoxification in *Escherichia coli*. Mol. Microbiol..

[7] Reiger M, Lassak J, Jung K (2015). Deciphering the role of the type II glyoxalase isoenzyme YcbL (GlxII-2) in *Escherichia coli*. FEMS Microbiol. Lett..

[8] Abdallah J, Mihoub M, Gautier V, Richarme G (2016). The DJ-1 superfamily members YhbO and YajL from *Escherichia coli* repair proteins from glycation by methylglyoxal and glyoxal. Biochem. Biophys. Res. Commun..

[9] Gautier V (2012). YajL, the prokaryotic homolog of the Parkinsonism-associated protein DJ-1, protects cells against protein sulfenylation. J. Mol. Biol..

[10] Wilson MA, Ringe D, Petsko GA (2005). The atomic resolution crystal structure of the YajL (ThiJ) protein from *Escherichia coli*: A close prokaryotic homologue of the Parkinsonism-associated protein DJ-1. J. Mol. Biol..

[11] Chakraborty S, Gogoi M, Chakravortty D (2015). Lactoylglutathione lyase, a critical enzyme in methylglyoxal detoxification, contributes to survival of Salmonella in the nutrient rich environment. Virulence.

[12] Habrych M, Rodriguez S, Stewart JD (2002). Purification and identification of an *Escherichia coli* beta-keto ester reductase as 2,5-diketo-d-gluconate reductase YqhE. Biotechnol. Prog..

[13] Ko J (2005). Conversion of methylglyoxal to acetol by *Escherichia coli* aldo-keto reductases. J. Bacteriol..

[14] Lee C (2010). Transcriptional activation of the aldehyde reductase YqhD by YqhC and its implication in glyoxal metabolism of *Escherichia coli* K-12. J. Bacteriol..

[15] Misra K, Banerjee AB, Ray S, Ray M (1996). Reduction of methylglyoxal in *Escherichia coli* K12 by an aldehyde reductase and alcohol dehydrogenase. Mol. Cell. Biochem..

[16] Gao X (2013). NleB, a bacterial effector with glycosyltransferase activity, targets GAPDH function to inhibit NF-kappaB activation. Cell. Host Microbe.

[17] Li S (2013). Pathogen blocks host death receptor signalling by arginine GlcNAcylation of death domains. Nature.

[18] Park JB (2018). Structural basis for arginine glycosylation of host substrates by bacterial effector proteins. Nat. Commun..

[19] Pearson JS (2013). A type III effector antagonizes death receptor signalling during bacterial gut infection. Nature.

[20] Brown NF (2011). Salmonella phage ST64B encodes a member of the SseK/NleB effector family. PLoS One.

[21] Ding J (2019). Structural and functional insights into host death domains inactivation by the bacterial arginine GlcNAcyltransferase effector. Mol. Cell..

[22] Esposito D (2018). Structural basis for the glycosyltransferase activity of the Salmonella effector SseK3. J. Biol. Chem..

[23] Newson JPM (2019). Salmonella effectors SseK1 and SseK3 target death domain proteins in the TNF and TRAIL signaling pathways. Mol. Cell Proteom..

[24] Gan J (2020). The Salmonella effector SseK3 targets small Rab GTPases. Front. Cell. Infect. Microbiol..

[25] El Qaidi S (2017). NleB/SseK effectors from *Citrobacter rodentium*, *Escherichia coli*, and *Salmonella enterica* display distinct differences in host substrate specificity. J. Biol. Chem..

[26] El Qaidi S (2020). An intra-bacterial activity for a T3SS effector. Sci. Rep..

[27] Hébrard M, Viala JPM, Méresse S, Barras F, Aussel L (2009). Redundant hydrogen peroxide scavengers contribute to *Salmonella* virulence and oxidative stress resistance. J. Bacteriol..

[28] O'Young J, Sukdeo N, Honek JF (2007). *Escherichia coli* glyoxalase II is a binuclear zinc-dependent metalloenzyme. Arch. Biochem. Biophys..

[29] Yang Z (2015). SseK3 is a salmonella effector that binds TRIM32 and modulates the host's NF-kappaB signalling activity. PLoS One.

[30] Chamchoy K (2020). Functional analysis of BPSS2242 reveals its detoxification role in *Burkholderia pseudomallei* under salt stress. Sci. Rep..

[31] Zhang MM, Ong CL, Walker MJ, McEwan AG (2016). Defence against methylglyoxal in Group A Streptococcus: A role for Glyoxylase I in bacterial virulence and survival in neutrophils?. Pathog. Dis..

[32] He MM, Clugston SL, Honek JF, Matthews BW (2000). Determination of the structure of *Escherichia coli* glyoxalase I suggests a structural basis for differential metal activation. Biochemistry.

[33] Stamp AL (2010). Structural and functional characterization of *Salmonella enterica* serovar Typhimurium YcbL: An unusual Type II glyoxalase. Protein Sci..

[34] Stebbins CE, Galán JE (2001). Maintenance of an unfolded polypeptide by a cognate chaperone in bacterial type III secretion. Nature.

[35] Wagner S (2018). Bacterial type III secretion systems: A complex device for the delivery of bacterial effector proteins into eukaryotic host cells. FEMS Microbiol. Lett..

[36] Krampen L (2018). Revealing the mechanisms of membrane protein export by virulence-associated bacterial secretion systems. Nat. Commun..

[37] Tucker SC, Galan JE (2000). Complex function for SicA, a *Salmonella enterica* serovar typhimurium type III secretion-associated chaperone. J. Bacteriol..

[38] Roe AJ (2003). Heterogeneous surface expression of EspA translocon filaments by *Escherichia coli* O157:H7 is controlled at the posttranscriptional level. Infect. Immun..

[39] Kujat Choy SL (2004). SseK1 and SseK2 are novel translocated proteins of *Salmonella enterica* serovar typhimurium. Infect. Immun..

[40] Baison-Olmo F, Galindo-Moreno M, Ramos-Morales F (2015). Host cell type-dependent translocation and PhoP-mediated positive regulation of the effector SseK1 of *Salmonella enterica*. Front. Microbiol..

[41] Qaidi SE, Hardwidge PR (2019). ABC cloning: An efficient, simple, and rapid restriction/ligase-free method. MethodsX.

[42] Datsenko KA, Wanner BL (2000). One-step inactivation of chromosomal genes in *Escherichia coli* K-12 using PCR products. Proc. Natl. Acad. Sci. USA.

[43] Rappsilber J, Mann M, Ishihama Y (2007). Protocol for micro-purification, enrichment, pre-fractionation and storage of peptides for proteomics using StageTips. Nat. Protoc..

[44] Cox J, Mann M (2008). MaxQuant enables high peptide identification rates, individualized p.p.b.-range mass accuracies and proteome-wide protein quantification. Nat. Biotechnol..

[45] Tyanova S (2015). Visualization of LC-MS/MS proteomics data in MaxQuant. Proteomics.

[46] Vizcaino JA (2016). 2016 update of the PRIDE database and its related tools. Nucleic Acids Res..

[47] El Qaidi S (2018). High-throughput screening for bacterial glycosyltransferase inhibitors. Front. Cell. Infect. Microbiol..

