# Supplementary material for: Arginine glycosylation enhances methylglyoxal detoxification
Source: Sci Rep. 2021 Feb 15;11:3834. doi: 10.1038/s41598-021-83437-0 (PMC7884692; doi:10.1038/s41598-021-83437-0)
Supplement: Supplementary file 1 — Supplementary Information 1. [file 41598_2021_83437_MOESM1_ESM.pdf]

Original images for Figure 2B

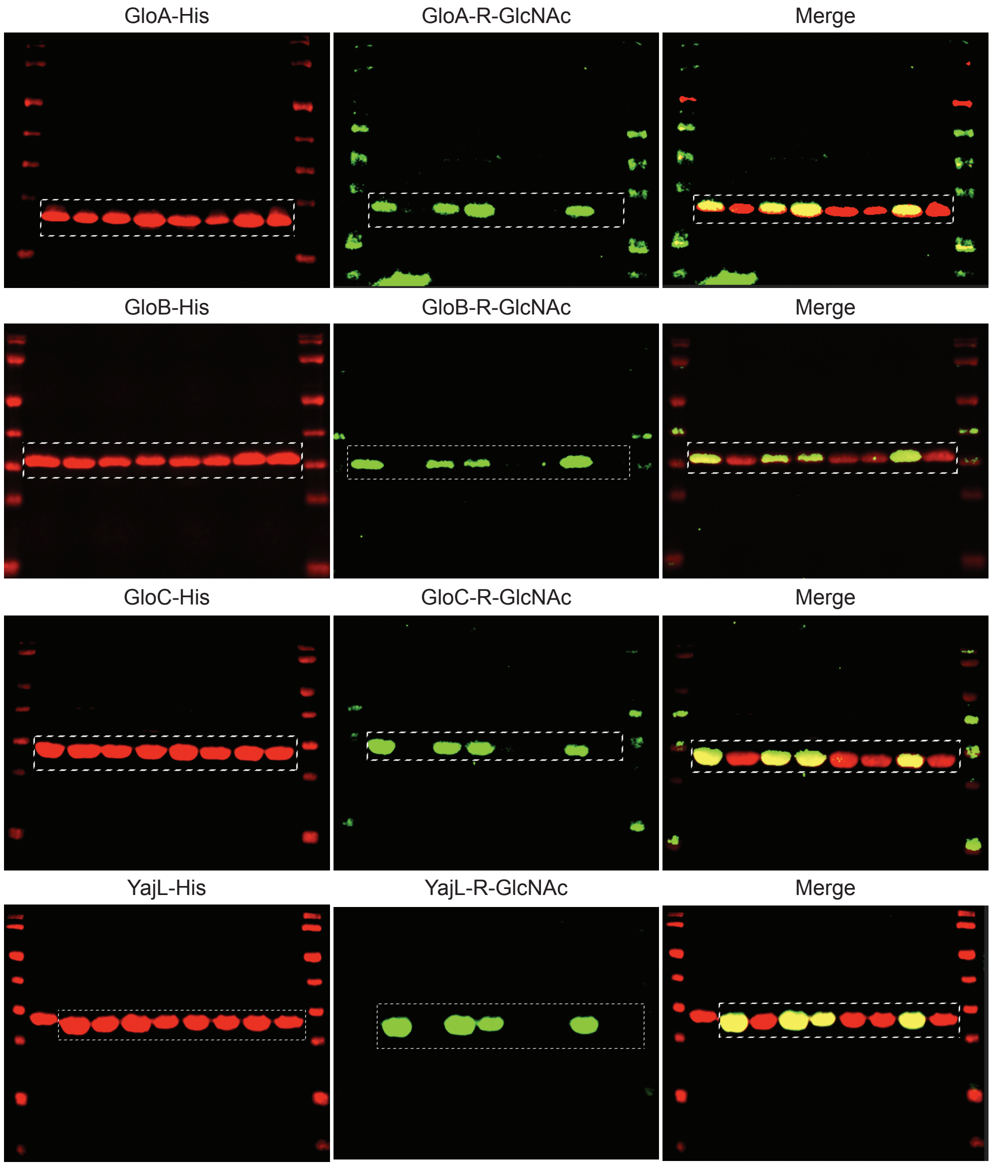

Note: YajL gels included an extra lane that was irrelevant to this study and was thus cropped from the images

Original images for Figure 4B

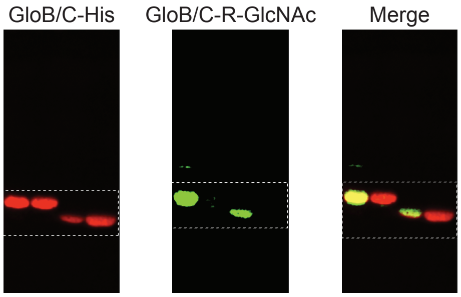

Original images for Figure 4C

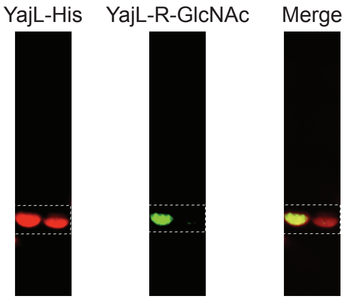

Original images for Figure 4D

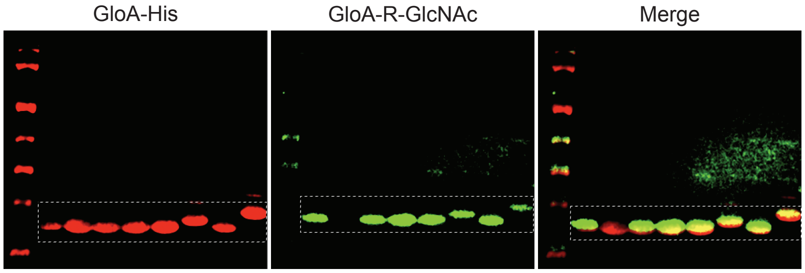

Supplemental Figure 1
