# Supplementary material for: Arginine glycosylation enhances methylglyoxal detoxification
Source: Sci Rep. 2021 Feb 15;11:3834. doi: 10.1038/s41598-021-83437-0 (PMC7884692; doi:10.1038/s41598-021-83437-0)
Supplement: Supplementary file 2 — Supplementary Information 2. [file 41598_2021_83437_MOESM2_ESM.docx]

**Supplementary Figures.**

**Supplemental Figure 1.** Original images for Western blots shown in Figures 2B, 4B, 4C, and 4D.
